# Methodological Shortcomings of Wrist-Worn Heart Rate Monitors Validations

**DOI:** 10.2196/10108

**Published:** 2018-07-02

**Authors:** Francesco Sartor, Gabriele Papini, Lieke Gertruda Elisabeth Cox, John Cleland

**Affiliations:** ^1^ Personal Health Philips Research Eindhoven Netherlands; ^2^ Department of Electrical Engineering Technical University Eindhoven Eindhoven Netherlands; ^3^ Institute of Health & Well-Being Robertson Centre for Biostatistics and Clinical Trials University of Glasgow Glasgow United Kingdom

**Keywords:** sensor technology, accuracy, wearable, telemonitoring

## Abstract

Wearable sensor technology could have an important role for clinical research and in delivering health care. Accordingly, such technology should undergo rigorous evaluation prior to market launch, and its performance should be supported by evidence-based marketing claims. Many studies have been published attempting to validate wrist-worn photoplethysmography (PPG)-based heart rate monitoring devices, but their contrasting results question the utility of this technology. The reason why many validations did not provide conclusive evidence of the validity of wrist-worn PPG-based heart rate monitoring devices is mostly methodological. The validation strategy should consider the nature of data provided by both the investigational and reference devices. There should be uniformity in the statistical approach to the analyses employed in these validation studies. The investigators should test the technology in the population of interest and in a setting appropriate for intended use. Device industries and the scientific community require robust standards for the validation of new wearable sensor technology.

In the past 5 years, there has been a huge proliferation of wrist-worn heart rate monitors, often embedded in smart-bands and smartwatches, which can generate a vast amount of data on lifestyle, physiology, and disease providing exciting opportunities for future health applications. Wearable sensor technology could have an important role for clinical research and in delivering health care [1]. Wearable sensors can be used to encourage healthier living (possible delaying or preventing the onset of disease), screen for incident disease, and provide unobtrusive continuous monitoring for people with chronic illnesses in order to optimize care and detect disease progression and complications. In Figure 1, we show an overview of potential continuous heart rate monitoring applications. New diagnostic applications could become possible thanks to the integration of heart rate and personal information such as age, sex, fitness, activity type, and symptoms. A large number of lifestyle apps and games are emerging thanks to continuous heart rate monitoring, currently most of them related to fitness (eg, Google Fit, Strava) or biofeedback relaxation (eg, Letter Zap, Skip a Beat). It is conceivable that health-promoting apps or games based on heart rate will soon be developed. Wearable heart rate monitors could also enable therapeutic monitoring such as medication titration. Accordingly, such monitors should undergo rigorous evaluation prior to market launch, and their performance should be supported by evidence-based marketing claims [1].

There are several types of validation studies. These studies may be marketing claim validations or medical claim validations for medical grade certification. They are usually done by the manufacturers, sometimes in collaboration with clinical sites, on unreleased products. There may also be benchmarking validation studies, where several commercially available competing products are compared to one another and against a reference. In some cases, there may be even single device validation studies.

**Figure 1:**
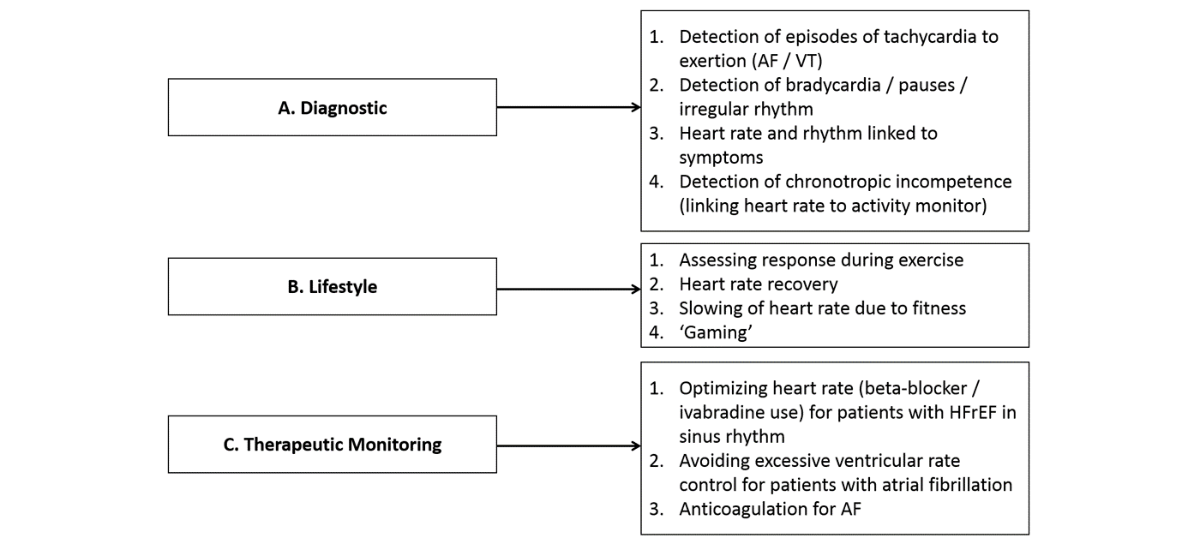
Brief overview of potential clinical and nonclinical applications derivable from continuous heart rate monitoring. AF/VT: atrial fibrillation/ventricular tachycardia; HFrEH: heart failure with reduced ejection fraction.

The latter 2 types are generally performed by academic or clinical centers even though industries often engage in such comparisons as well. The only studies which go through a strict quality regulatory framework are medical claim validation studies for medical grade certification (eg, Food and Drug Administration in the United States, medical CE [Conformité Européene] marking in Europe) [2,3]. As a consequence, many nonmedical devices are released on the market without rigorous validation.

In Europe, the choice on how to position a device is the responsibility of the manufacturer, whereas in the United States, this decision can be overruled if the device is perceived to have potential health risks for the user [4]. Because manufacturers can decide whether or not they wish to comply with medical certification regulations, this inevitably leads to heterogeneity in what validations are done. In our view, the lack of stringent regulations for the release of nonmedical heart rate monitoring devices should not justify the lack of standard requirements for validating this technology. The adoption of such technology by health care professionals could be hampered by their liability in case of adverse events when using commercially available nonmedical devices. The authors of this viewpoint agree with Quinn [4], who suggests “a more pragmatic, risk-based approach,” which takes a case-by-case look at commercial solutions that may or may not meet the standards required of medical devices. This approach should be applied to promote technology adoption and at the same time safeguard the safety of end-users. Here, we give an overview of clinical applications exploiting wearable heart rate monitors.

In a Research Letter recently published in JAMA [5], the performance of several commercially available, wrist-worn photoplethysmography (PPG)-based heart rate monitors was reported. The authors concluded that PPG-based monitoring was not suitable “when accurate measurement of heart rate is imperative.” The authors of that Research Letter acknowledged their report had limitations, including testing only 1 type of activity (treadmill), only in healthy people, and noncontinuous monitoring. Many other studies have been published validating wrist-worn PPG-based heart rate monitoring devices [6-14] but fail to show consensus in favor of or against the accuracy of this sensing technology.

The authors believe that the reason why many validations did not provide conclusive evidence of the validity of wrist-worn PPG-based heart rate monitoring devices is mostly methodological. Studies conducted by teams with a biomedical engineering background are more concerned with addressing problems like signal synchronization and averaging, while research teams with a sports medicine background are more concerned with target groups and exercise protocols. Moreover, clinicians are primarily interested in apps related to telemonitoring, in-hospital or remote. Each approach has its methodological shortcomings. The aim of this viewpoint is to suggest a more consistent and robust approach to validating monitoring technologies.

When validating heart rate monitoring devices, it is sensible to follow a common definition of accuracy. The American National Standards Institute standard for cardiac monitors, heart rate meters, and alarms defines accuracy as a “readout error of no greater than ±10% of the input rate or ±5 bpm, whichever is greater” [15]. Once accurate heart rate is defined, it is also good to agree on what to use as a gold standard. Electrocardiography (ECG) is the accepted gold standard for heart rate monitoring. Nevertheless, ECG, as with PPG, can be severely affected by artifacts [16]. Yet it is generally accepted that PPG-based heart rate monitoring suffers from inherent drawbacks (eg, more difficult peak detection, higher sensitivity to motion artifacts) compared to ECG-based monitoring [16].

The validation strategy should consider the nature of data provided by investigational devices (ID) and reference devices (RD). Heart rate values are always derived from more complex signals (eg, ECG, PPG). Thus, even when the ID and RD have the same output rate (eg, 1 heart rate value per second) and these outputs are well synchronized, the beats compared may not belong to the same time intervals. The method used to extract information from the raw data (eg, time domain or frequency domain) and the averaging strategy (eg, interbeat intervals or 5-second periods) of the raw data will determine a specific time lag for each heart rate value. Ideally, researchers should have access to the raw data. This is often not possible, and it should be acknowledged as a limitation.

Researchers should realize that their RD (often an ECG device) will not always be accurate. Unless there is a quality check on the validity of the ECG, a second reference device should be used such as a second ECG-based sensor applied in a different manner (eg, patch versus chest strap) and using a different software algorithm for calculating heart rate. When the two RDs fail to agree, no comparison should be made between RD and ID outputs (Figure 2). As mentioned earlier, even the RD (for example ECG patch or ECG strap) in certain circumstances may suffer from inaccuracy due to artifacts (eg, motion artifacts). Based on our own experience in testing hundreds of subjects, we realized that ECG patches perform particularly badly when the skin under the electrodes is stretched or excessively wet. ECG straps perform rather poorly when the skin gets too dry, the strap loosens up, and for certain anatomical shapes (pectus excavatum). These problems must be reported by the researcher.

**Figure 2:**
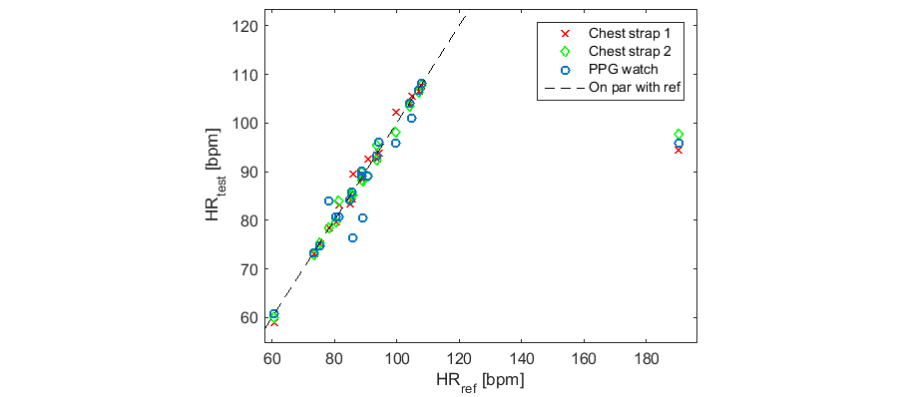
Correlation between 3 heart rate (HR) monitoring devices and the electrocardiography (ECG) reference. When the 2 chest straps and the wrist-worn photoplethysmography (PPG) heart rate monitors consistently disagree with the reference, their points depart from the 45-degree line in the same way.

**Figure 3:**
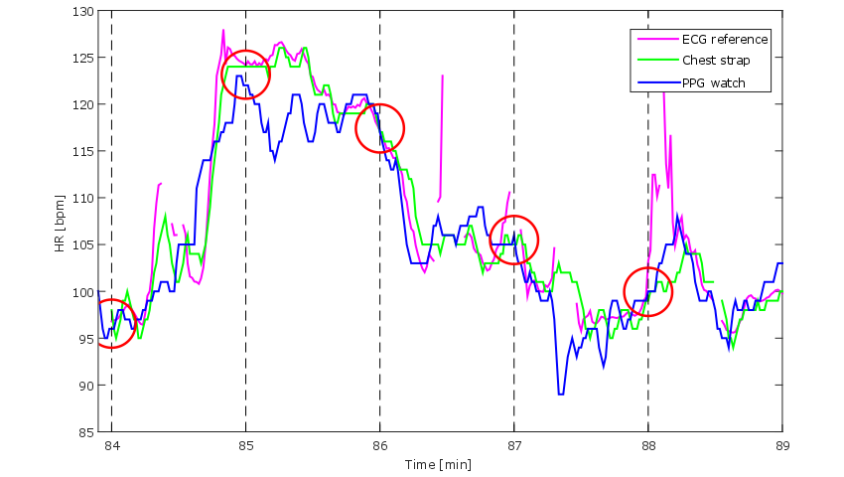
Segment of heart rate (HR) recordings by 3 devices: electrocardiography (ECG) reference, chest strap, and photoplethysmography (PPG) watch. The red circles represent the instants when heart rate from those devices would be collected if these were a value per minute observation. It is evident how these values do not represent the actual second by second or even the average agreement among the 3 devices.

The accuracy of the observation method should be robust (ie, repeatable and reproducible). In some validation studies, heart rate was logged manually after visually consulting the display of both ID and RD [5,7]. This method carries several limitations including human data entry errors and failure to report precisely simultaneous values from multiple devices. This method also limits the observation rate to, for instance, 1 value per minute [5,6]. Taking 1 value per minute is not the same as taking an averaged value over a minute, and both approaches fail to capitalize on the information derived from the rates of change in heart rate and heart variability and assume that participants are in a steady-state condition. Researchers should choose the observation rate (eg, 1 or 5 values per second) and averaging strategy (eg, 5- or 30-second windows) according to the use case foreseen for the heart rate monitor. Yet researchers need to be aware that taking, or averaging, 1 value every minute will hide variability [17]. This is evident in Figure 3, which illustrates that 1 single time point (red circles) is not necessarily representative of the entire minute. Consequently, for the purpose of testing accuracy, even when a mean heart rate value per minute would be sufficient, accuracy should be evaluated at the highest resolution possible.

We also observed a lack of uniformity in the statistical analyses employed in validation studies. Pearson correlations and Student *t* tests are inadequate for testing agreement [18]. This is because the Pearson correlation coefficient is not sensitive to systematic deviations from the 45-degree line, failing to reject agreement when these deviations occur. The Student *t* test is inadequate in rejecting agreement when means are equal but the 2 measures do not correlate with each other, and it can reject agreement when a very small systematic residual error shifts 1 of the means [19]. Moreover, the *t* test assesses difference, which implies that when not rejecting the null hypothesis (ie, means are equal) it does not prove that the 2 means are equivalent. Concordance correlation coefficients should be reported instead [18,19]. Also, limits of agreement analyses should be accompanied by typical error calculations [20]. Equivalence testing should be used when the alternative hypothesis is that the outputs of 2 devices are the same [21]. In equivalence testing, the null hypothesis is that the differences between the means are outside the equivalence limits.

Finally, there are some practical considerations. The investigators should test the technology in the population of interest and in a setting appropriate for intended use. Measurements taken at rest or in the period after exercise cannot be considered to validate measurements done during exercise. Results gathered on healthy individuals with no abnormal heart rhythm are inappropriate for applications aimed at patients with cardiovascular disease where the burden of arrhythmias will be substantially higher. Additionally, due to the effect that the contact of the sensor with the skin and the environmental conditions can have on the PPG signal, information such as sensor placement, strap tightness, skin type, temperature, and possibly light intensity should be reported.

Although many studies have been published to assess the validity and usability of wrist-worn PPG-based heart rate monitoring, their methodological differences and shortcomings hamper research into their clinical utility and their introduction into health care. Such devices could make an important contribution to the future of mobile health and, in our view, should be rigorously evaluated as outlined above. For the reasons discussed in this viewpoint, we advocate standard requirements generally accepted by both the scientific community and the device industries in order to provide a fair and consistent validation of new wearable sensor technology.

## Abbreviations

ECG: electrocardiography
ID: investigational device
PPG: photoplethysmography
RD: reference device
